# Human Immune Responses to *H. pylori* HLA Class II Epitopes Identified by Immunoinformatic Methods

**DOI:** 10.1371/journal.pone.0094974

**Published:** 2014-04-16

**Authors:** Songhua Zhang, Joseph Desrosiers, Jose R. Aponte-Pieras, Kristen DaSilva, Loren D. Fast, Frances Terry, William D. Martin, Anne S. De Groot, Leonard Moise, Steven F. Moss

**Affiliations:** 1 Division of Gastroenterology, Rhode Island Hospital, Warren Alpert Medical School of Brown University, Providence, Rhode Island, United States of America; 2 Institute for Immunology and Informatics, University of Rhode Island, Providence, Rhode Island, United States of America; 3 Division of Hematology and Oncology, Rhode Island Hospital, Warren Alpert Medical School of Brown University, Providence, Rhode Island, United States of America; 4 EpiVax, Inc., Providence, Rhode Island, United States of America; University of Aberdeen, United Kingdom

## Abstract

*H. pylori* persists in the human stomach over decades and promotes several adverse clinical sequelae including gastritis, peptic ulcers and gastric cancer that are linked to the induction and subsequent evasion of chronic gastric inflammation. Emerging evidence indicates that *H. pylori* infection may also protect against asthma and some other immune-mediated conditions through regulatory T cell effects outside the stomach. To characterize the complexity of the CD4+ T cell response generated during *H. pylori* infection, computational methods were previously used to generate a panel of 90 predicted epitopes conserved among *H. pylori* genomes that broadly cover HLA Class II diversity for maximum population coverage. Here, these sequences were tested individually for their ability to induce in vitro responses in peripheral blood mononuclear cells by interferon-γ ELISpot assay. The average number of spot-forming cells/million PBMCs was significantly elevated in *H. pylori*-infected subjects over uninfected persons. Ten of the 90 peptides stimulated IFN-γ secretion in the *H. pylori*-infected group only, whereas two out of the 90 peptides elicited a detectable IFN-γ response in the *H. pylori*-uninfected subjects but no response in the *H. pylori*-infected group. Cytokine ELISA measurements performed using in vitro PBMC culture supernatants demonstrated significantly higher levels of TNF-α, IL-2, IL-4, IL-6, IL-10, and TGF-β1 in the *H. pylori*-infected subjects, whereas IL-17A expression was not related to the subjects *H. pylori*-infection status. Our results indicate that the human T cell responses to these 90 peptides are generally increased in actively *H. pylori*-infected, compared with *H. pylori*-naïve, subjects. This information will improve understanding of the complex immune response to *H. pylori*, aiding rational epitope-driven vaccine design as well as helping identify other *H. pylori* epitopes with potentially immunoregulatory effects.

## Introduction

*Helicobacter pylori (H. pylori)* is a NIAID emerging pathogen [1] that infects the gastric mucosa of half the human population, leading to chronic gastric inflammation in all and clinically important adverse outcomes in a sizable minority. While most infections with this gram-negative bacterium are not associated with symptoms, gastric or duodenal ulcers ultimately develop in approximately 10% of colonized individuals, with gastric adenocarcinoma or mucosal-associated lymphoid tissue lymphoma occurring in about 1%, decades after the initial infection. *H. pylori* infection is unevenly distributed, being most prevalent in resource-poor countries (in the range of 70–90%) and in as few as 10% or less of some Western populations [2]. Stomach cancer, which is largely attributable to *H. pylori* [3], is responsible for 10,900 deaths per year in the US [4] and about 738,000 deaths annually worldwide [5]. *H. pylori* infection, usually acquired in childhood, leads to the recruitment of immune and inflammatory cells to the stomach. [2] The pathogenesis of gastritis, peptic ulcer disease and gastric cancer associated with *H. pylori* is subsequently linked with the intensity and quality of the innate and adaptive chronic immune responses in the gastric mucosa. [6], [7]

Although infection with *H. pylori* is pathogenic in a subset of the colonized population, “beneficial” effects of persistent *H. pylori* infection have been proposed, due to a consistent inverse relationship between the loss of gastric *H. pylori* colonization and the emergence of certain conditions of increasing prevalence in fully industrialized nations, including some characterized by unrestrained inflammation outside the stomach. These conditions include childhood-onset asthma, inflammatory bowel disease, eosinophilic esophagitis and even esophageal adenocarcinoma. [8], [9], [10], [11], [12] Experimental evidence in mice suggests that the acquisition of *H. pylori* infection early in life may reprogram mucosal and systemic immunity in the direction of increased regulatory T cell function and decreased inflammation outside the stomach [13], [14].

Because interactions between *H. pylori* and the immune system are implicated in both the pathogenesis of *H. pylori*-associated diseases and “protection” against certain extra-gastric conditions, it is important to understand precisely how *H. pylori* interacts with elements of the host immune system. We have used computational methods to identify a core *H. pylori* genome, comprising 676 open reading frames from seven genetically and phenotypically diverse *H. pylori* strains [15]. The core genome served as a source for identification of HLA Class II epitopes with broad coverage of circulating *H. pylori* strains. Complementing *H. pylori* coverage, we used immunoinformatic tools to identify 90 T-cell epitopes that broadly cover HLA Class II diversity for maximum population coverage (see **Table S1**). HLA binding studies validated computational predictions with 79% accuracy for a panel of six HLA Class II alleles, representing >90% of the global human population [15]. Here, we report functional studies testing the human T cell responses to each of these 90 peptides by interferon-γ ELISpot assay, since this is a widely used screening method to measure effector functions of low-frequency antigen-specific T cell populations to class II epitopes in human PBMCs [16] including to a putative *H. pylori* vaccine candidate. [17]

This is the first large-scale discovery study for HLA class II-restricted *H. pylori* immune epitopes. Prior studies focused on HLA class II epitopes found in the urease beta subunit and in neuraminyllactose-binding hemagglutinin. [17], [18] Using an unbiased approach we have probed the entire *H. pylori* proteome to identify T cell targets within conserved consensus and potentially immunogenic peptide sequences. Our results indicate that these sequences generally elicited significantly higher in vitro responses in those patients already infected by *H. pylori* in comparison with *H. pylori*-naïve subjects. This information will improve understanding of the complex immune response to *H. pylori* and should aid rational epitope-driven vaccine design.

## Results

### *H. pylori* peptides predicted by immunoinformatic screening to be T-cell epitopes are recognized preferentially by *H. pylori*-infected patients PBMC's in interferon-γ ELISpot assays

**Figure 1:**
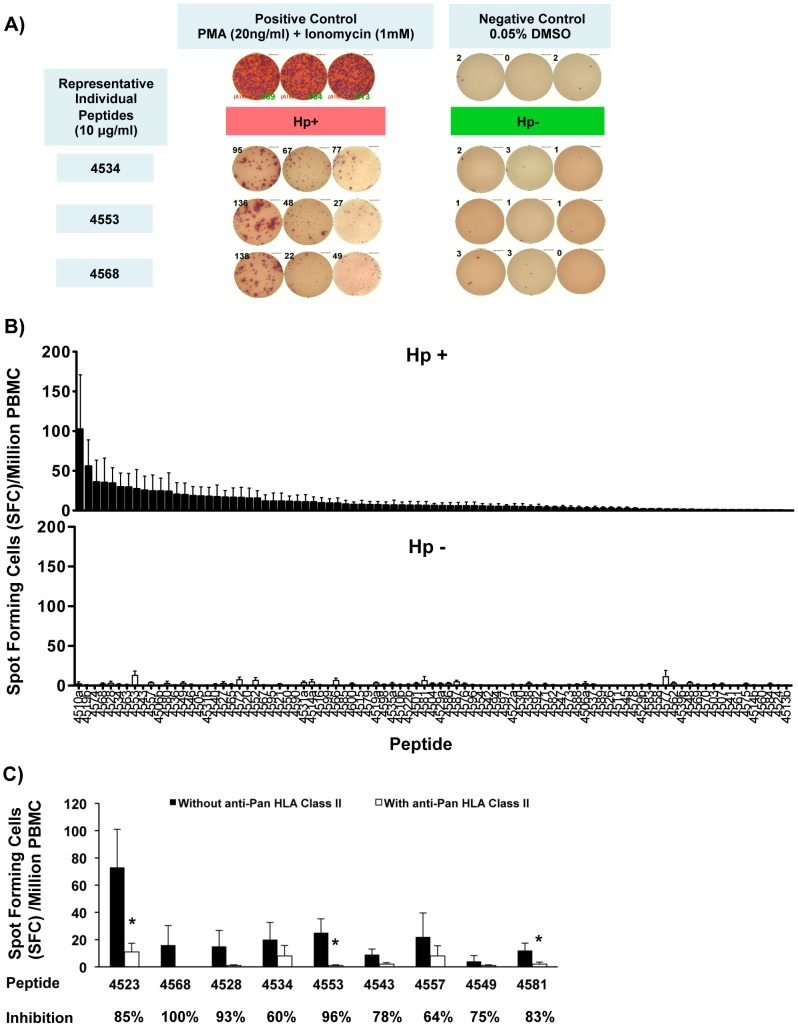
In vitro PBMC IFN-γ secretion measured by ELISpot in response to 90 individual *H. pylori* genome-derived peptides. A) **Representative human IFN-γ ELISpot images. Representative images of IFN-γ ELISpot we**lls stimulated by three different epitopes (4534, 4553 and 4568). Data are from three patients in each group. The number of spots per well is stated by the side of each well image. *H. pylori* genome-derived peptides are recognized preferentially by PBMCs from *H. pylori*-infected patients. Stimulation with Phorbol 12-myristate 13-acetate (PMA) (20 ng/ml) and ionomycin (1 mM) provide the positive control, while 0.05% dimethyl sulfoxide (DMSO) treated PBMCs served as a negative control in this assay. Hp+: *H. pylori*-infected patients, Hp-: *H. pylori*-uninfected patients. **B**) **ELISpot results for each peptide sorted from highest spot forming cell number to lowest in the** ***H. pylori*** **-infected subjects.** PBMCs extracted from 15 *H. pylori*-infected and 15 *H. pylori*-uninfected patients were co-cultured with 90 individual *H. pylori* peptides (10 µg/ml) in anti-human IFN-γ pre-coated 96-well ELISpot plates for 24 hours. IFN-γ spot forming cells (SFC) were counted by an S5 automated immunospot analyzer. Each column represents the average of SFC per million PBMCs over 2x background for each peptide in *H. pylori*-infected subjects + SEM (Hp+, black column, top panel) and *H. pylori*-uninfected subjects (Hp-, white columns, lower panel), respectively. P<0.0001, *H. pylori*-infected versus uninfected group overall. **C**) **Inhibition of peptide-stimulated IFN-γ production by Pan-HLA II blocking antibody.** PBMCs from 8 *H. pylori*-infected subjects were co-cultured with the peptides shown in the absence (black columns) or presence (white columns) of Pan-HLA II blocking antibody. Data expressed as the average (+SEM) of SFC per million PBMCs over 2x background with mean % inhibition due to the blocking antibody listed below each peptide number. * P<0.05, absence vs presence of blocking antibody.

To test the immunogenicity of a panel of 90 peptides predicted by immunoinformatic screening to contain conserved *H. pylori* epitopes, human IFN-γ ELISpot assays were performed in 96-well plates pre-coated with human IFN-γ specific monoclonal capture antibodies. PBMC (2.5×10^5^/well) isolated from *H. pylori-*infected and uninfected patients were incubated with 90 *H. pylori* antigen-specific peptides individually at a final concentration of 10 µg/ml for 24 hours. Representative ELISpot images from three *H. pylori*-infected and three *H. pylori*-uninfected patients are shown in **Figure 1A**, demonstrating the increased number of IFN-γ secreting cells in *H. pylori*-infected subjects.

In general, the majority of the 90 peptides elicited a higher IFN-γ ELISpot response (in terms of number of spot forming cells) in *H. pylori*-infected subjects than in the uninfected subjects (**Figure 1B**). Using as a threshold for positivity the 2x background vehicle value, the mean (± SEM) number of SFC per million PBMCs across all subjects and across all peptides was 10.8±1.5 in the *H. pylori*-infected versus 1.9±0.2 in the uninfected cases (*** P<0.0001). The individual responses of each subject to each peptide tested are listed in **Table S2**.

Ten of the 90 peptides stimulated IFN-γ secretion in the *H. pylori*-infected group only, whereas two out of the 90 peptides elicited a weak IFN-γ response in the *H. pylori*-uninfected subjects but no response in the *H. pylori*-infected group.

The specificity of the response was verified by blocking class II HLA presentation with a pan- HLA class II antibody. The 9 most frequently recognized peptides from the 90 peptide panel were evaluated in the 8 most highly responsive *H. pylori* infected subjects in the presence or absence of the blocking antibody. The results indicate that the blocking antibody suppressed the mean IFN-γ ELISpot response in all patients for each of the peptides tested (**Figure 1C**), and overall by an average of 81.6%, P<0.01.

### *H. pylori* genome-derived peptides elicited strong in vitro Th1/Treg, but not Th17A cytokine expression by PBMCs from *H. pylori*-infected humans, when compared to *H. pylori*-uninfected group

**Figure 2:**
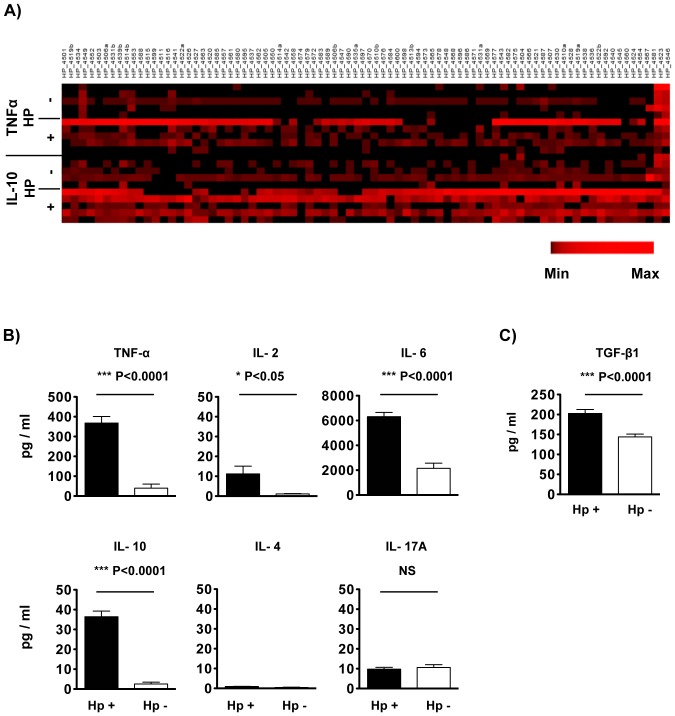
In vitro PBMC cytokine profiling in ELISpot well supernatants in response to 90 individual *H. pylori* genome-derived peptides. Supernatants from ELISpot assay cultures were assayed for multiple cytokine concentrations by cytometric beads array (CBA) and TGF-β1 concentration by ELISA. (A) Individual peptide responses for individual patients are shown in heatmap format for TNF-α (Th1) and IL-10 (Treg) cytokines. The heat map rows represent individual patients (5 *H. pylori* positive and 5 *H. pylori* negative) and the columns represent responses to individual peptides. Graphical analysis of the dataset was performed using PermutMatrix 1.9.3 [51]. For comparison of different cytokines, expression levels were normalized. (B, C) Columns represent average values (± SEM) of 90 individual peptide-stimulated cytokine secretion minus background values in *H. pylori*-infected patients (Hp+, white column) and *H. pylori* uninfected patients (Hp-, black column). All data are shown as specific cytokine concentration in pg/ml of supernatant (mean ± SEM). * P<0.05; *** P<0.0001; NS: no significant difference; N = 5.

To further evaluate T cell responses to each of these peptides, ELISpot well supernatants from a subset of five patients in each group (*H. pylori*-infected and uninfected) were assayed by a multiplex cytokine bead array. The five patients per group were selected based on their being the most responsive to the peptide panel in ELISpot analysis. All the data were expressed as mean ± SEM after subtracting background values (DMSO vehicle controls). Individual peptide responses for all five patients in each group illustrate elevated Th1 and Treg cytokine responses in *H. pylori*-infected patients (**Figure 2A**). Overall, averaging the data for all 90 peptides, there was significantly higher expression of TNF-α (368±33.5 versus 40±20.5 pg/ml, ***P<0.0001), IL-2 (11.21±3.9 versus 1.1±0.1 pg/ml), IL-6 (6314±35.1 versus 2151±418.9 pg/ml, ***P<0.0001) and IL-10 (36.5±2.8 versus 3±0.9 pg/ml, ***P<0.0001) in the supernatants from *H. pylori*-infected subjects compared to *H. pylori*-uninfected subjects, respectively. (**Figure 2B**). Furthermore, there was a statistically significant correlation between TNF-α expression and IFN-γ ELISpot results (P<0.0001, R^2^ = 0.2217). IL-4 levels were extremely low in both groups of patients and IL-17A levels were not significantly different between the *H. pylori*-infected versus uninfected subjects (9.8±0.9 versus 10.6±1.4 pg/ml). Individual level results per patient per peptide are listed in **Table S3**.

TGF-beta 1 (TGF-β1) protein expression was analyzed in the same 5 supernatants from each group by ELISA (for individual level results per patient per peptide see **Table S3**). As shown in **Figure 2C**, TGF-β1 expression in PBMC supernatants from peptide-stimulated wells was higher in the *H. pylori*-infected subjects than the uninfected subjects (202.8±9.9 versus 144.0±7.3 pg/ml, ***P<0001), which is consistent with the IL-10 profile above and the immunosuppressive role these cytokines play in maintaining *H. pylori* persistence and mediating *H. pylori*-specific immunomodulation [19]. However, the difference between groups for TGF-β1 was relatively small, and because there is an acid activation step used to measure TGF-β1 by ELISA, the levels may not reflect accurately differences in the biologically active protein (reviewed in [20]).

### Cytokine correlations with epitope mapping predictions

The relationship between computational predictions of *H. pylori* HLA Class II ligands made by the EpiMatrix epitope mapping algorithm [15] and their recognition by PBMCs as experimentally tested here was determined. Because HLA types are diverse in the human population, the predictive profile of the epitope peptide sequences was initially determined for each individual subject according to the subject's HLA type [21]. The predictive profile is described by an individual T cell epitope measure (iTEM) value that is calculated per sequence as an estimate of the likelihood that it would stimulate an immune response, as described in the Methods. iTEM values ranged from the lowest value for a single prediction in the top 5^th^ percentile of EpiMatrix prediction for a single DR allele, 1.65, up to 12.21, representing a sequence with multiple hits for two different DR alleles of a subject.

**Figure 3:**
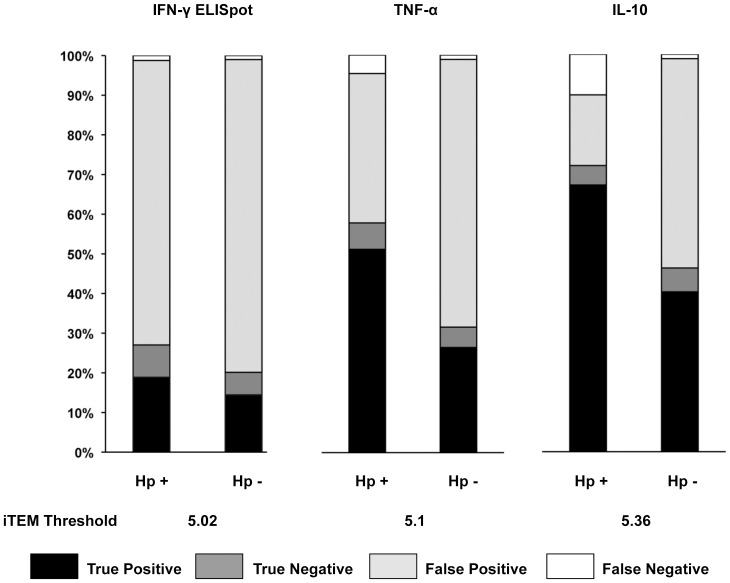
Comparison of T cell epitope predictions and cytokine responses to 90 individual *H. pylori* genome-derived peptides. Per subject and per peptide, the predictive accuracy of IFN-γ ELISpot assay responses (N = 15 per cohort) and TNF-α and IL-10 CBA assay responses (N = 5 per cohort) was determined according to subject HLA type, as described in the Methods and Results. Stacked bars illustrate the proportions of true and false positive predictions and true and false negative predictions per cohort and per cytokine.

Correlations were then made between iTEM values and the Th1 and Treg cytokine data for all predicted epitopes across the *H. pylori*-infected and uninfected cohorts (**Table S4**). Th1 and Treg cytokines were selected for analysis for their importance in *H. pylori* immunity and the statistically significant distinction between *H. pylori*-infected and uninfected subject responses to the predicted epitopes observed for these cytokines. A minimal iTEM value that predicts cytokine stimulation with statistical significance was determined, as described in the Methods, for *H. pylori*-infected subjects, the population expected to respond to the peptides. For IFN-γ measured by ELISpot assay, the iTEM value is 5.02 (N = 15); for TNF-α, 5.10, and IL-10, 5.36, as measured by the CBA assay (N = 5). The iTEM algorithm predicts with high sensitivity (>87% for all cytokines), as the proportion of true positives to false negatives is high, whereas specificity is low because of high numbers of false positives (**Figure 3**). False positives arise, in part, because of error in predictions, and, in part, due to (i) low frequencies of antigen-specific T cells, which are to be expected in subjects who were likely initially infected with *H. pylori* decades before this study, (ii) leukocytes taken from outside the primary site of infection, and due to (iii) the type of cytokine measurements performed in this study where cells were not cultured to expand low frequency clones. We note that the proportion of false positives is noticeably lower for TNF-α and IL-10 than IFN-γ, even for the IFN-γ dataset from the subset of five subjects used to measure TNF-α and IL-10 (data not shown). This may be related to the difference in defining a positive response in the assays used to measure these cytokines: whereas bulk cytokine secretion above background is generally accepted to be significant in the CBA assay, in the ELISpot assay cytokine-producing cells exceeding twice background levels are considered significant. Alternatively, iTEM may be a better predictor of amount of cytokine production than numbers of cytokine-producing cells, but that could be reliably determined only by comparing the two assays for production of the same cytokine, which was not feasible in this study. Finally, we note that when the iTEM values above are applied to the respective cytokine datasets collected from *H. pylori*-negative subjects, it is apparent that the proportion of true positive and negative predictions in infected subjects is noticeably greater than in uninfected, as expected.

## Discussion

We observed antigen-specific functional differences between the T cell repertoires of *H. pylori*-positive and -negative subjects for a set of 90 immunoinformatic-predicted T-cell epitopes from seven genetically diverse *H. pylori* strains. This study expands the number of known *H. pylori* epitopes that stimulate human T cell responses. A search of the Immune Epitope Database reveals only 11 peptides from three different antigens discovered until now [17], [18], [22], [23] The present study uncovered 90 new epitopes derived from 81 unique antigens.

Many *H. pylori* gene products have been tested for vaccine development in preclinical studies over the last three decades since *H. pylori* was recognized. However, the only vaccine candidates that have been evaluated in human subjects are whole formalin-inactivated *H. pylori*, [24] urease subunits A and B (heterodimers necessary for urease's catalytic activity), [25], [26], [27] HP0231 (function currently unknown) [26] and a trivalent vaccine comprising recombinant CagA (cytotoxin-associated gene A), VacA (vacuolating cytotoxin A) and NAP (neutrophil activating protein). [28] Of the 81 proteins represented by our 90 peptide panel, most are related to metabolic and intracellular signaling pathways [15] and none has been previously suggested for consideration as an *H. pylori* vaccine candidate or been identified as a correlate of *H. pylori* immunity. Interestingly, among the predicted epitopes in the current study three are related to *H. pylori's* flagellum (flagellum-specific ATP synthases, flagellar biosynthesis protein FlhA and flagellar basal body L-ring protein), and the urease accessory protein ureH that functions with three other urease accessory proteins (urease E, F and G) to regulate the insertion of nickel to activate the urease A/B complex. [27] Furthermore, among the 90 computationally-selected peptides in our panel, 5 are related to *H. pylori's cag* pathogenicity island, a 32-gene cluster associated with *H. pylori* virulence (ulcer disease and cancer) that encodes a type IV secretion system capable of inserting bacterial products (such as the CagA protein) into host epithelial cells. [29] CagA has been extensively studied as a putative bacterial oncoprotein and as an immunodominant and long-lived marker of past and current *H. pylori* infection [29]. Two of the predicted epitopes in our panel were in Cag6 (also known as CagZ or HP0526 in the *H. pylori* strain 26695 nomenclature), a high-abundance protein essential for CagA translocation, [30] and others were in Cag11 ( = CagU = HP0531, an inner membrane protein essential for secretion apparatus function [31]), Cag17 ( = CagN = HP0538, a poorly characterized periplasmic component of the type IV secretion system [30] and HP1451 (an inhibitor of HP0525 ( = Cag alpha) which is an inner membrane ATPase essential for CagA translocation. [32] Thus several peptide sequences discovered by our informatics screen relate to proteins known to be essential for *H. pylori* virulence, including flagellar function (motility), urease activity (acid tolerance) and the *cag* pathogenicity island (type IV secretion).

The fact that any one specific peptide only elicits a response in on average 20.2 % of *H. pylori*-infected subjects suggests that a multi-epitope approach for *H. pylori* vaccine development will be needed when using a computational approach to epitope prediction.

Importantly, the results also suggest that extragastric T cells with *H. pylori* specificities form an antigen-specific memory T cell repertoire in the periphery in *H. pylori*-positive humans. Cytokine responses are (i) observed only when stimulated by peptides, (ii) are mediated by HLA class II presentation as anti-Class II antibody blocks peptide-stimulated IFN-γ responses and (iii) are detected in brief overnight cultures following blood collection. Surprisingly, similar, though weaker and lower frequency, responses in the *H. pylori*-naïve population were detected. As a primary response under in vitro assay conditions is unlikely, these *H. pylori*-specific responses may represent T cells educated on cross-reactive antigens from commensals or other pathogens. A deeper understanding of these heterologous immune responses may shed light on how some *H. pylori* naïve persons are protected from disorders associated with the absence of *H. pylori*.

Recently, a model relating how *H. pylori* infection stimulates antigen presenting cells to program CD4+ T cells has emerged from mouse studies [33]. It proposes that *H. pylori* drive gastric dendritic cells (DCs) to become tolerogenic. These DCs migrate to mesenteric lymph nodes where they encounter naïve T cells and convert them to Tregs, while poorly inducing Th1 and Th17 cells [34]. Relative amounts of IL-18 and IL-1β in the DC/T cell microenvironment determine whether Treg/Th1 or Th17 cells are preferentially induced. We observed increased Th1 and Treg signature cytokine production (TNF-α and IL-10) in *H. pylori*-infected subjects in multiplex cytokine assays of peptide-stimulated in vitro PBMC cultures. Th1 cytokine stimulating sequences also elicit immunosuppressive cytokine production. These results underscore the complexities of *H. pylori*-stimulated T cell responses. While the phenotype of the cell that secretes IL-10 has not yet been determined, the result is consistent with human studies that uncovered gastric Treg-produced IL-10 in *H. pylori* infection [35]. Whatever the phenotype, these results provide a unique opportunity to begin to relate specific *H. pylori*-derived sequences to extragastric immunity that is believed to be responsible for the growing list of benefits of early childhood *H. pylori* infection.

It is also thought that weak TLR signaling in infection results in a lack of IL-1β production, which is needed to drive Th17 differentiation [36]–[39]. *H. pylori* LPS and flagellin are poor activators of their respective TLRs 4 and 5, in comparison with their counterparts in other gram negative bacteria [40], [41], [42]. Moreover, while *H. pylori* bears TLR2 ligands, they stimulate largely anti-inflammatory responses in vivo [43]. As a result, IL-1β levels are more limiting than IL-18, so that IL-18 dominates and Th17 differentiation stalls. As a measure of Th17 stimulation, we measured IL-17A elicited by epitope peptides and found that levels were not elevated in T cells from *H. pylori*-infected relative to uninfected patients. However, the time course of IL17 secretion is usually delayed compared with other T-cell dependent cytokines after antigen stimulation [44], which may partly explain the lack of difference at the single, 24-hour timepoint that we measured. Additionally, the RPMI medium that we used in the ELISpot assay is not optimal for Th17 polarization since it has relatively low levels of the aromatic amino acids necessary for activation of the aryl hydrocarbon receptor, unlike Iscove's modified Dulbecco's medium, for example. [45] Nonetheless, we note that both IL-6 and TGF-β1, which are critical for Th17 differentiation [46], are elevated in *H. pylori*-infected subjects.

The development of a preventive or therapeutic vaccine against *H. pylori* continues to be elusive [47], [48]. An *H. pylori* vaccine is recognized to be especially valuable for resource-poor countries with a high burden of *H. pylori*-related disease, especially as antibiotic-resistant strains are becoming more prevalent globally [49], [50]. On the other hand, a putative beneficial effect of *H. pylori* on asthma and possibly other allergic and inflammatory conditions outside the stomach in fully industrialized nations demonstrates the need to better understand the complex interactions of *H. pylori* with the human immune system when developing vaccines or other *H. pylori* immunotherapeutics. The epitopes identified in this study may serve as tools to characterize *H. pylori*-specific T cells involved in cross-suppression of allergy or chronic inflammation or activation of inflammatory T cells in humans, thereby aiding the development of novel immunotherapy and vaccine strategies, respectively, against this persistent and ubiquitous gastric bacterium. The phenotypes, frequencies and avidities of these distinct T cells are properties that may influence beneficial or detrimental responses to subsequent antigenic stimulation in vivo and are therefore important to further characterize in future studies.

## Materials and Methods

### Ethics statement

Informed written consent for the study was obtained from all patients before they underwent endoscopy under a protocol approved by Rhode Island Hospital's Institutional Review Board and in accordance with the principles expressed in the Declaration of Helsinki. No children were recruited into the study.

### Subjects and *H. pylori* determination

Subjects were recruited for the present study from patients age ≥18 years undergoing esophagogastroscopy at Rhode Island Hospital. Exclusion criteria were active gastrointestinal bleeding, anemia, pregnancy, previous gastric surgery, use of nonsteroidal anti-inflammatory drugs (NSAIDs), anticoagulant drugs, proton pump inhibitors (PPI), histamine (H2)-receptor antagonists, bismuth compounds or antibiotics within the previous 1 month, and endoscopic findings of malignant or bleeding lesions. *H. pylori* status was ascertained by histology (hematoxylin and eosin staining), immunohistochemistry and rapid urease testing of gastric biopsies.

A total of 15 *H. pylori*-infected and 15 *H. pylori*-uninfected patients were recruited. Their average age was 48±2.9 years. 53.3 % were male, 16.7% were Asian, 40.0% were Hispanic or Latino and 43.3 % were white. There were no significant differences between the groups for age, gender or racial/ethnic distributions.

### Peripheral blood mononuclear cells (PBMCs) extraction

Approximately 60 ml of peripheral venous blood was obtained by venipuncture from each subject and PBMCs harvested using lympholyte H (Cedarlane Laboratories USA Inc., Burlington, NC) as the density gradient according to the manufacturer's instructions. After washing with PBS, PBMCs were resuspended in RPMI 1640 supplemented with 10% heat inactivated human AB serum (Valley Biomedical Products & Services, Inc. Winchester, VA), 100 U/ml penicillin, 100 µg/ml streptomycin and 2 mM L-glutamine (Lonza, Hopkinton, MA). Viable cells were counted by trypan blue exclusion, and 2.5×10^5^ cells per well were immediately plated for ELISpot assays with the peptide panel. Approximately 5×10^6^ PBMCs per subject were stored at −80°C for HLA typing.

### Epitope selection and peptide synthesis

The top-scoring 90 *H. pylori* consensus sequences, according to predictive epitope mapping using the EpiMatrix algorithm and in vitro HLA binding assays [15], were synthesized as 15∼25 mer peptides (mean length 19-mer). Synthetic peptides were manufactured using 9-fluoronylmethoxycarbonyl (Fmoc) chemistry by 21st Century Biochemicals (Marlboro, MA). Peptide purity was >80% as ascertained by analytical reversed phase HPLC. Peptide mass was confirmed by tandem mass spectrometry. Individual peptides were dissolved in sterile dimethyl sulfoxide (DMSO) and used at a final concentration of 10 µg/ml. **Table S1** lists the identity of the peptide sequences that we tested and their corresponding proteins using the nomenclature of *H. pylori* reference strain 26695, from which the sequences were derived, as described previously [15].

### Enzyme-linked immunosorbent spot (ELISpot) assay

Freshly extracted PBMCs were pipetted at 2.5×10^5^/well into 96-well multiscreen HTS filter plates (EMD Millipore, Billerica, MA) for human IFN-γ ELISpot assay (eBioscience Inc., San Diego, CA). Each well was stimulated for 24 hours with 10 µg/ml of a single peptide. Triplicate controls per subject were: DMSO 0.05% as the negative control and 20 ng/ml Phorbol 12-myristate 13-acetate (PMA) with 1 mM ionomycin (Sigma-Aldrich, St. Louis, MO) as the positive control. Inhibition of peptide-stimulated IFN-γ production was performed on frozen PBMCs in the presence or absence of anti-HLA DP, DR, DQ antibody (Clone: Tu39, BD Biosciences). IFN-γ spot forming cells (SFC) were counted by an S5 automated immunospot analyzer (Cellular Technology Limited, Shaker Heights, OH). Average IFN-γ responses to each peptide were calculated after subtraction of two times the background IFN-γ immunospots obtained from the mean of the negative control wells. Peptide elicited IFN-γ responses were expressed as spot forming cells (SFC) /million PBMCs.

### Characterization of the secreted cytokine profile

Twenty-four hour PBMC supernatants from each ELISpot well were collected from five *H. pylori*-infected and five *H. pylori* uninfected subjects and assayed for TNF-α, IL-10, IL-6, IL-4, IL-2 and IL-17A using the human Th1/Th2/Th17A CBA assay kit (BD Biosciences, San Jose, California) on an LSR II flow cytometer (BD Biosciences). From these same 24-hour PBMC supernatants TGF-β1 was measured by an ELISA (eBioscience, San Diego, CA) that has a detection range of 8 to 1000 pg/ml.

### Human leukocyte antigen (HLA) typing

Donor HLA Class II types were determined using the Micro SSPTM High Resolution HLA Class II kit (One Lambda, Canoga Park, CA) at the Hartford Hospital Transplant Immunology Laboratory.

### Statistical analysis of immunoassay data

All statistical evaluation was performed with GraphPad Prism version 5.01 (GraphPad, La Jolla, CA). The SFC comparison and difference of cytokine expression between the two groups (*H. pylori* positive versus negative) was done by unpaired student t test with Welch's correction. The correlation between SFC and TNF-α expression was evaluated by Pearson correlation coefficient. P values of <0.05 were considered to be statistically significant.

### Immunoinformatic analysis

Per subject and per predicted epitope sequence, individual T cell epitope measure (iTEM) scores were calculated by summing top 5^th^ percentile EpiMatrix Z-scores (hits) according to subject HLA type over both DR alleles [21]. For sequences containing multiple hits, per allele, scores in descending magnitude were weighted by a multiple of 1/x for ‘x’ number of hits.

Per subject, iTEM scores for the set of 90 peptides were correlated with ELISpot and CBA assay data for each cytokine. The minimal iTEM score that positively correlates with cytokine data with statistical significance (p<0.05) by the Chi-squared test for the *H. pylori*-infected cohort across all epitopes tested was calculated. To determine this value, iTEM score and cytokine data pairs were classified as true or false positives or true or false negatives. True positives are peptide-HLA pairs with both positive iTEM scores and cytokine values. True negatives are peptide-HLA pairs with both negative iTEM scores and cytokine values. False positives have positive iTEM scores and negative cytokine values, and false negatives, have negative iTEM scores and positive cytokine values. For cytokine data, the cutoff for positivity was positive values greater than twice background levels in the ELISpot assay and positive values after background subtraction in the CBA assay. The iTEM value determined for the *H. pylori*-infected cohort was applied to the *H. pylori*-negative dataset for comparison of the true and false positives and true and false negatives of the two groups.

## Supporting Information

Table S1 ***H. pylori*** **peptides used in PBMC studies.** PEPTIDE ID refers to a four-digit identifier for each peptide. 26695 ACCESSION NUMBER refers to the GenBank accession number source of the initial 9-mer “seed” for each predicted *H. pylori* Class II peptide. SEQUENCE refers to the amino acid sequence of the given peptide. SOURCE PROTEIN refers to the protein description from which each predicted epitope is derived.
(XLSX)

Table S2 **IFN-γ ELISpot results listed by subject and peptide.** HLA types are listed as shown below each subject's designation. All values for peptide responses are the number of spot forming cells per million after subtraction of two times the background values obtained from the mean of the negative control wells.
(XLSX)

Table S3 **Cytokine bead array and TGF-β1 results listed by subject and peptide.** Each tab of the spreadsheet opens a data sheet for an individual cytokine. HLA types are listed as shown below each subject's designation. Subject identity numbers correspond to the subjects listed in Table S2.
(XLSX)

Table S4 **Accuracy of predicted cytokine responses listed by subject and peptide.** Each tab of the spreadsheet opens a data sheet for an individual cytokine. Subject identity numbers correspond to the subjects listed in Table S2. HpN =  *H pylori* negative subject; HpP  = *H pylori* positive subject. PEPTIDE NAME refers to a four-digit identifier for each peptide. PEPTIDE SEQUENCE refers to the amino acid sequence of the given peptide. TEST OUTCOME refers to the spot number or cytokine concentration measured by ELISpot or CBA assay, respectively. Values above zero are defined as positive responses, as described in the Methods and Results. ALLELE 1/ALLELE 2 EPIMATRIX MAX SCORE refers to the EpiMatrix Z-score of the highest scoring 9-mer sequence of a peptide. Cutoff for positivity is Z-score ≥1.64 (top 5^th^ percentile). EPIMATRIX PREDICTION STATUS refers to predictive accuracy of cytokine response in relation to both EpiMatrix max scores (true or false positive, true or false negative). ITEM SCORE refers to the iTEM score calculated for the combined HLA class II alleles of the subject for a given peptide. Cutoffs for positivity (described in Results): IFN-γ ELISpot: 5.02; TNF-α CBA: 5.1; IL-10 CBA: 5.36; IL-2: 2.4; IL-4: 8.71; IL-6: 5.0; IL-17A: 7.5. ITEM PREDICTION STATUS refers to predictive accuracy of cytokine response in relation to iTEM score (true or false positive, true or false negative).
(XLSX)
